# Super-Resolution Imaging Strategies for Cell Biologists Using a Spinning Disk Microscope

**DOI:** 10.1371/journal.pone.0074604

**Published:** 2013-10-09

**Authors:** Neveen A. Hosny, Mingying Song, John T. Connelly, Simon Ameer-Beg, Martin M. Knight, Ann P. Wheeler

**Affiliations:** 1 Blizard Institute, Barts and the Royal London School of Medicine and Dentistry, Queen Mary University London, London, United Kingdom; 2 Blizard Institute, Barts and the Royal London School of Medicine and Dentistry, Queen Mary University London, London, United Kingdom; 3 Randall Division of Cell and Molecular Biophysics, Kings College London, London, United Kingdom; 4 School of Engineering and Materials Science, Queen Mary University London, London, United Kingdom; University of California, Berkeley, United States of America

## Abstract

In this study we use a spinning disk confocal microscope (SD) to generate super-resolution images of multiple cellular features from any plane in the cell. We obtain super-resolution images by using stochastic intensity fluctuations of biological probes, combining Photoactivation Light-Microscopy (PALM)/Stochastic Optical Reconstruction Microscopy (STORM) methodologies. We compared different image analysis algorithms for processing super-resolution data to identify the most suitable for analysis of particular cell structures. SOFI was chosen for X and Y and was able to achieve a resolution of *ca.* 80 nm; however higher resolution was possible >30 nm, dependant on the super-resolution image analysis algorithm used. Our method uses low laser power and fluorescent probes which are available either commercially or through the scientific community, and therefore it is gentle enough for biological imaging. Through comparative studies with structured illumination microscopy (SIM) and widefield epifluorescence imaging we identified that our methodology was advantageous for imaging cellular structures which are not immediately at the cell-substrate interface, which include the nuclear architecture and mitochondria. We have shown that it was possible to obtain two coloured images, which highlights the potential this technique has for high-content screening, imaging of multiple epitopes and live cell imaging.

## Introduction

Many biological structures are too small to be resolved by standard confocal microscopy, which has a limit of resolution of approximately 200 nm. Until recently this has restricted the scope of research into small molecular structures and cellular complexes, such as bacteria, viruses, membrane vesicles, nuclear ultrastructure and cytoskeletal filaments. Super-resolution microscopy has circumvented this resolution limit, described by Abbe's law [1], [2], permitting observations of structures as small as 30 nm in size. This technological revolution advances our understanding of molecular cell biology as it reveals novel biological phenomena at nanometre resolution.

Super-resolution techniques can be performed by a number of approaches [3]; Structured Illumination microscopy (SIM) is a technique where a grid pattern, generated from diffraction of light, is super-imposed on the specimen and rotated in steps. The output dataset is processed with specialised algorithms giving an improvement in lateral resolution by a factor of two [4]. Other techniques, such as STED, GSD, SSIM, PALM, STORM, FPALM, dSTORM, GSDIM and PAINT rely upon the principles of Reversible Saturable Optical Fluorescence Transitions (RESOLFT) microscopy. In RESOLFT proteins or organic fluorophores are switched between dark and fluorescent states stochastically, data are captured and processed to give an output image with resolution refined beyond the Abbe limit of 200 nm [2], [3], [5].

Methods of super-resolution which use stochastic molecular switching, do not require specialised microscopy systems [5]; instead they generate super-resolved images by iteratively activating a set of photo-switchable fluorophores and precisely fitting the point of emission through complex image analysis [6], [7]. Techniques such as photo-activation light microscopy (PALM) [6], stochastic optical reconstruction microscopy (STORM) [8] and ground state depletion microscopy (GSDIM) [9], [10] all operate on the statistical methods principle. PALM uses photo-switchable fluorescent proteins and STORM/GSDIM photo-switchable fluorescent dyes to generate stochastic fluorescent emissions which are imaged and then processed to refine image resolution [5]. The methodologies employed by image processing algorithms for statistical SR methods fall broadly into two categories; specific identification of spatially separate individual fluorescent emission events and fitting of these events in an reconstructed image, e.g. RainSTORM [11], QuickPALM [12] and GLRT [13] or higher order statistical analysis of intensity fluctuations e.g. SOFI [14], [15], 3B [16], Deconvolution-STORM (DeconSTORM) [17] and Faster-STORM [18] (Table S1). These latter group of image analysis methodologies, such as SOFI and Decon-STORM do not require single molecule activations and were developed for super-resolution imaging of structures which may be more densely labelled by fluorescent dyes [15], [17]. For all RESOLFT methods using iterative imaging of stochastic light emission the photon yield of the dye, detector sensitivity and detector resolution play a key role in determining the level of resolution improvement that can be obtained [19].

PALM, STORM and other forms of statistical super-resolution methodology require only a standard light microscope and electron-multiplying charge-coupled device (EMCCD) camera as hardware [5]. For these techniques to work well, image datasets must be acquired at fast frame rates and with good signal-to-noise (S/N). This ensures a sufficient number of stochastic fluorescent emission events are collected for the super-resolution image analysis algorithms to work accurately [1]. It can be challenging to obtain good S/N in PALM/STORM using biological samples in widefield illumination. This is due to photo-bleaching of the fluorophore labelling the epitope of interest, which reduces the number of fluorophores actively emitting light; and artefacts arising from photo-interactions above and below the focal plane. The out of focus light is generated from fluorescent emission of labelled proteins that are not in the desired plane of focus and the viscous nature of the cytoplasm, which scatters light [5]. These photo-interactions, from above and below the focal plane, impede correct reconstruction of the super-resolution image.

Total Internal Reflection Microscopy (TIRF) overcomes these issues by creating an evanescent wave that only illuminates a thin (<100 nm) optical section at the immersion oil and coverslip interface omitting out of focus light [20]. The excellent S/N this achieves makes TIRF the standard method for PALM and STORM imaging. Unfortunately, TIRF visualisation is not appropriate for all biological samples due to the limited imaging depth [20]. This means that structures further away from the coverslip than 100 nm such as; the cells' nucleus and organelles immediately surrounding the nucleus such as the endoplasmic reticulum, mitochondria and Golgi apparatus, are cannot be imaged using TIRF based PALM/STORM. Recent research has tried to overcome this problem by using a TIRF microscope with double objectives to visualise the actin cytoskeleton at the very top and the bottom of the cell [21]. Selective plane illumination microscopy (SPIM) has also been used to generate 3D super-resolution images [22]. Another alternative is ‘near TIRF’ where a highly inclined laminated optical light sheet (HILO) is generated using an intense laser illumination of light, angled through a high numeric aperture objective [23]. However, HILO only increases the depth of light penetration into the cell to 500 nm, so cellular structures which are 2–3 µm inside the cell such as the nucleus, Golgi apparatus, Endoplasmic reticulum and mitotic spindle still cannot be visualised.

**Figure 1:**
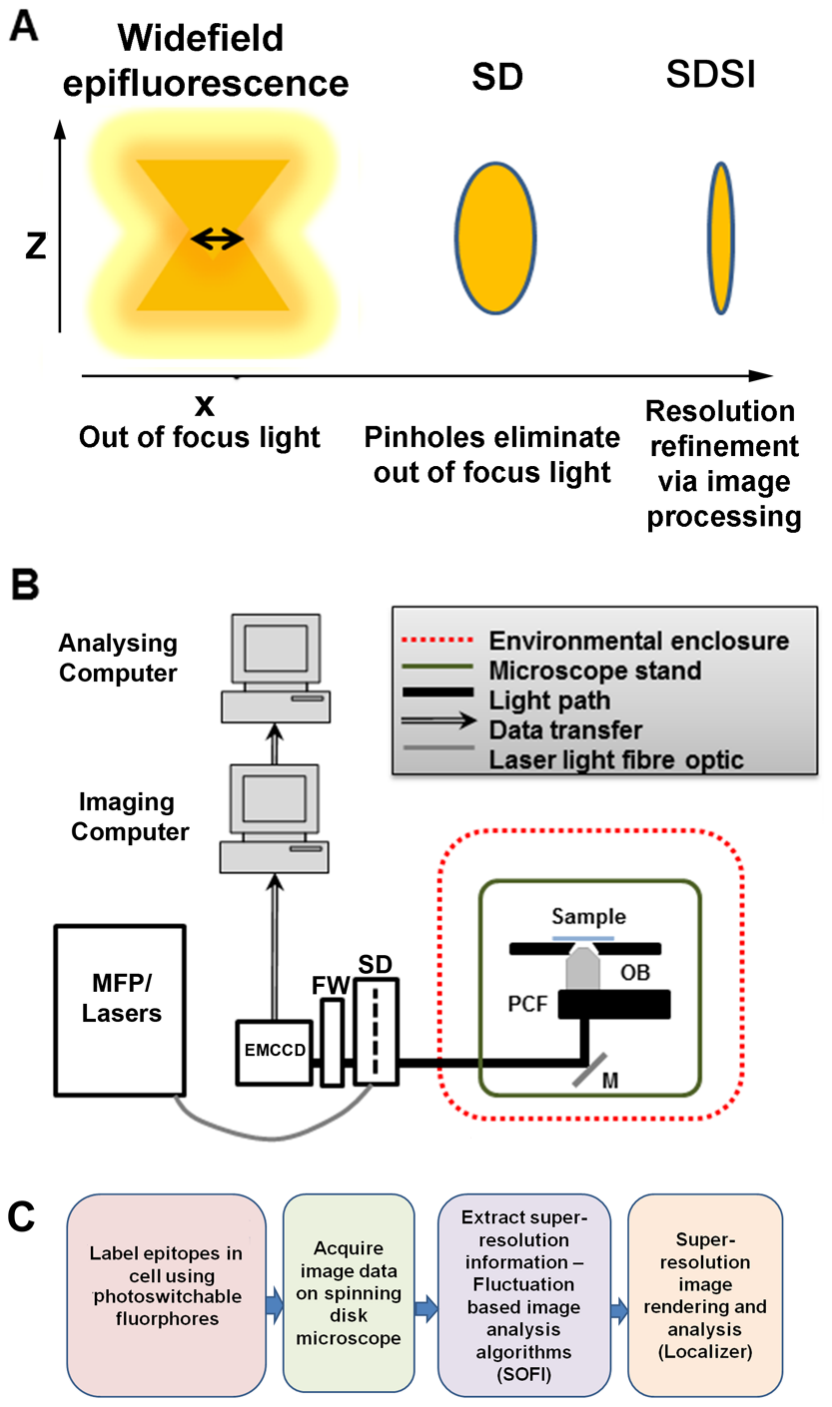
Setup of spinning disk stochastic imaging (SDSI) system and evaluation of imaging capabilities. (A) Schematic diagram describing how the point spread function is refined in a selected axial plane by spinning disk confocal microscopy and super-resolution image processing. (B) Diagram showing the configuration of the SDSI microscope, abbreviations are as follows. EMCCD = Electron multiplied Charge Coupled Device camera. FW = Filter wheel. SD = Yokagawa CSUX1 spinning disk, M = mirror. PCF = Piezo coupled focus feedback unit. OB = Objective. (C) Workflow of the SDSI experiments used in this paper, Briefly samples are prepared with probes for either PALM or dSTORM, next samples are imaged and finally SR data is generated using the SOFI image processing algorithm.

In widefield microscopy all of a specimen in the optical path of the microscope is excited by the light source. This means for a point source there will be in focus light and out of focus light present at the detector (Figure 1A).It is particularly important to improve the signal to noise ratio when imaging the nucleus at super-resolution as it is a dense structure in the centre of the cell and so a lot of out of focus light is present which degrades the quality and accuracy of the output image. Spinning disk confocal microscopy (SD) presents an excellent solution to this as it functions as a widefield confocal, selectively illuminating one focal plane with thousands of pinholes and omitting out of focus light (Figure 1A) [24]–[26]. The spinning disk speeds up the acquisition time, compared with a standard raster scanning confocal and improves the S/N ratio compared with a standard widefield epifluorescence. Combining the spinning disk together with super-resolution imaging allows a truly single plane super-resolution image to be acquired at any z-axis (Figure 1A), which allows any plane in the full depth of a cell to be imaged [25].

Here we combined the flexibility of an SD microscope with the simplicity of using PALM/STORM probes to present a novel methodology using spinning disk microscopy for super-resolution imaging (SDSI). We further present a comparative study of several image analysis algorithms available for PALM/STORM to inform potential users of the advantage different image analysis strategies provide for the interpretation of their particular datasets.

## Results

### Spinning disk super-resolution imaging set up

Spinning disk super-resolution imaging (SDSI) was developed to facilitate the super-resolution imaging of proteins or organelles at any single location in the cells axial (z) plane across a large field of view.

To implement SDSI it was necessary to perform the following modifications to our spinning disk set up (Materials and Methods, Figure 1B): Environmental vibrations were minimized by removing all non-essential equipment from the optical table and incorporating active damping legs to the optical bench (Figure 1A and Figure S1A–C) and isolating the system from air currents. This reduced system drift to around 40 nm reasonably consistently in SDSI experiments (Figure S1C). The accuracy of super-resolution image assignment is directly correlated to the number of photons detected and pixel size of the detector [6], [19] (materials and methods). The spinning disk unit (CSU) itself is moderately light inefficient [26], [27] therefore laser power was increased slightly above levels normally used in routine SD imaging to maximise sufficient photon counts. We investigated different detectors for SDSI and identified that an EMCCD camera with an 70% quantum efficiency and 8 µm pixels was required, as a minimum, to detect sufficient photons for the super-resolution image reconstruction on our system using a 100× 1.4NA objective. This is because the number of photons detected and pixel size of the detector determine the amount of resolution refinement as explained in the equation given by Thompson et al [19] (materials and methods). We also found it necessary to use fluorescent probes with high quantum efficiency for data reconstruction, and confirmed that TdEos, mEOS, Dronpa, AlexaFluor555 and AlexaFluor647 performed well in our experiments.

**Figure 2:**
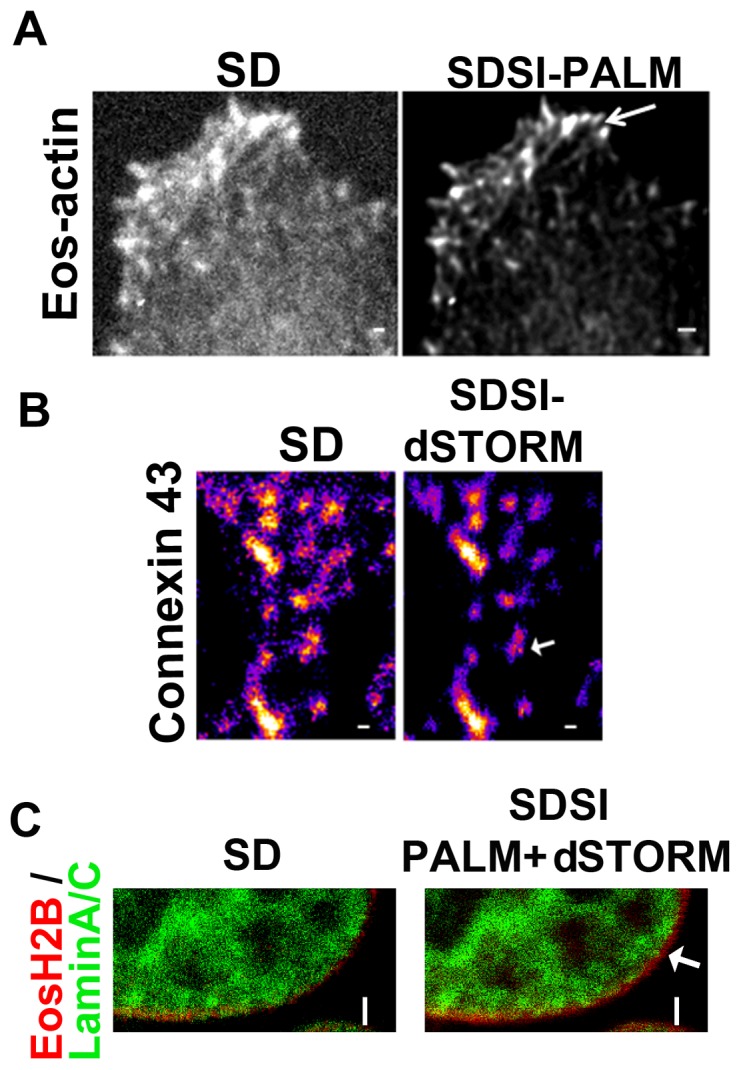
SDSI super resolution imaging using both PALM and STORM. (A) Comparison of SD and PALM images (processed by SOFI) of Eos-Actin, (B) Comparison of SD and d-STORM images of Connexin 43, Secondary Fab fragment antibodies conjugated to AlexaFluor647 were used for dSTORM imaging, SR data was generated using 3^rd^ order SOFI, in both images bar = 1 µm. (C) Comparison of SD and SDSI combining PALM and dSTORM imaging of Eos-Histone 2B (PALM) and Lamin A/C (dSTORM). Secondary Fab fragment antibodies conjugated to AlexaFluor647 were used for dSTORM imaging. SR data was generated using 3^rd^ order SOFI. Arrow indicates individual histone complexes, bar = 2 µm.

As proof of principle, we performed experiments that visualised cellular structures smaller than the resolution limit of a standard confocal microscope. We also examined two different super-resolution imaging methodologies PALM and STORM to determine if both of these techniques could be used with SDSI. Eos Actin was used to generate PALM images of the actin structure in the lamellipodia of cells (Figure 2A). The Eos Actin dataset was also used to confirm and optimise the occurrence and collection of photo-switching events (Figure S2A and B). Antibodies were used to generate STORM images of Connexins trafficking through the cell (Figure 2B). PALM required cells to be transfected with a photo-convertible probe that switched between the off and on state through illumination from two lasers. To determine if PALM could work with SDSI, HeLa cells were transfected with tdEos-Actin. Actin was imaged using simultaneous imaging of the sample with an activation laser at low power (405 nm laser, 1–5 mW) and an imaging laser at medium power (561 nm laser, 10–12.5 mW). Over the course of the experiment the laser power of both the activation and imaging laser were modulated to ensure only a sparse population of fluorophores were present in each frame (Figure 2A and B). dSTORM microscopy relies on a combination of standard chemical dyes and a bespoke image buffer that is used to reactivate dyes, which are in the fluorescent off-state (i.e. not emitting light but not photo-bleached) (Materials and Methods) [10]. For STORM microscopy the composition of the imaging buffer was modified to account for the dye used [18]. To visualise Alexa Fluor 647 labelled Connexin vesicles cells were again imaged carefully by monitoring the laser power (640 nm laser, 8–16 mW). Laser power was altered to ensure a sparse set of fluorophores were present and that photo-bleaching of the AlexaFluor647 was minimised to ensure sufficient signal was available for image reconstruction (Figure 2B) [10]. All SDSI images were processed using the 3^rd^ order SOFI algorithm implemented in the Localizer software suite [13], [14] achieving a minimum resolution of 80 nm (Figure S3A). Two colour super resolution imaging was conducted by combining photoswitchable fluorescent proteins and dyes combining PALM and dSTORM sample preparation methodologies, by transfecting cells with Eos-Histone 2B fluorescent proteins (PALM) and LaminA/C labelled with AlexaFluor647 (dSTORM) (Figure 2C). The presence of the buffer for dSTORM did not perturb photo-conversion events for Eos fluorescent proteins (Figure 2C). For correct image registration of two colour data was correctly 100 nm gold beads were added to the sample as fiduciary marks and left to settle onto the glass. The electrostatic charge on the glass was sufficient to hold the beads in place during imaging. The gold beads were visible in all imaging channels, we also found that 40 nm gold beads could be used if a higher level of resolution accuracy was required. Taken together these data show that both PALM and dSTORM sample preparation methodologies can be used separately and together to acquire super-resolved data on a spinning disk microscope.

### SDSI image analysis

**Figure 3:**
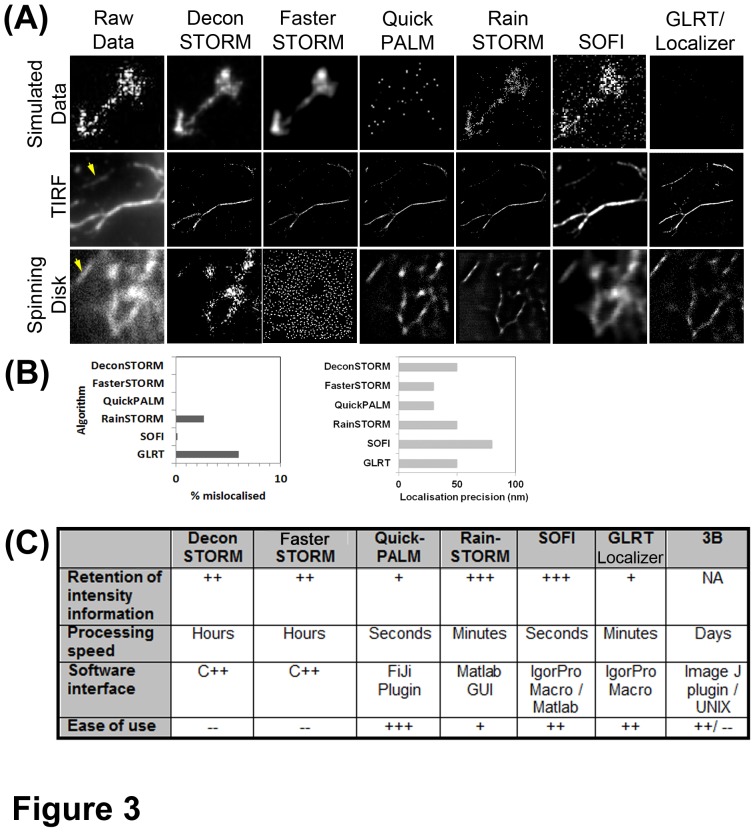
Comparative study of different stochastic super-resolution image processing algorithms. (A) Reconstructed super-resolution images from: a simulated dataset of 800 frames, a 64×64 pixel, 5000 frame dSTORM dataset of actin filaments labelled with AlexaFluor647 visualised by TIRF microscopy, a 90×90 pixel 5000 frame PALM dataset of Hela cells transfected with Eos-Actin. SR images were generated using DeconSTORM, FasterStorm, QuickPALM, RainSTORM, 3^rd^ order SOFI and GLRT implemented in the Localizer image analysis package respectively. An image of the raw, unreconstructed, data is shown for comparison. For TIRF and SD this is the average of 8 frames of data, for the simulated data a maximum intensity projection of the whole dataset is shown. (B) Graphs showing the percentage of mislocalized pixels in datasets reconstructed from a noisy background image. The noisy background image was generated by acquiring 5000 frames of images of a red fluorescent Perspex slide. Graph showing the predicted localization precision between all of the investigated algorithms. (C) Chart comparing retention of intensity information, processing speed, software interface and ease of use of the software of all the algorithms in the comparative study.

All super-resolution data reconstruction algorithms require a certain minimum number of photo-conversion events to correctly assign structure. Under-sampling of super-resolution data can lead to artefacts where the structure is not completely assigned. To find the best image analysis methodology for SDSI comparative studies between seven different algorithms, (Table S1 and Materials and Methods) were conducted. Three separate datasets were used: a simulated dataset of overlapping emitters [17] (Movie S1) this dataset was chosen to model data from our biological system as it comprises simulated overlapping emission events. A dataset generated using recombinant actin filaments visualised using TIRF (Movie S2) (a kind gift from D.Metcalf, NPL, UK), and Eos-actin filaments in cells generated by SDSI (Movie S3). Analysis of the reconstructed simulated data showed the single molecule fitting algorithms QuickPALM and GLRT could not reconstruct the simulated dataset, this is because it contained overlapping emitters (Figure 3A). Of the algorithms that reconstructed the simulated dataset, FasterSTORM and DeconSTORM gave smoothened results, whereas RainSTORM and SOFI produced results more representative of the sample data (Figure 3A). We also performed comparative analysis with a noisy background sample. We found GLRT, and RainSTORM would on occasion mis-assign the background as a positive signal (Figure 3B). The spatial fitting for both TIRF and SR data varied between the algorithms with QuickPALM and FasterSTORM reported to give the highest accuracy (Figure 3B) [12], [18]. However, visual analysis of both the TIRF and SDSI images showed that only SOFI and RainSTORM reconstructed all of the features in the original image (Figure 3A). The partial reconstruction of images is likely due to either overlapping emitters, which the algorithms rejects (Table S1), or a low number of stochastic emitting events occurring in the region reconstructed during data collection; collecting more data could remedy the latter problem. The TIRF dataset did appear to be slightly better resolved; most likely due to more photo-conversion events being detected by TIRF than SDSI due to light inefficiency of the spinning disk or because of increased sensitivity of the camera on the TIRF system (Figure 3A and Figure S3B). This may explain why Deconvolution STORM processed TIRF data well and the SDSI data poorly. FasterSTORM was not able to process SDSI images (Figure 3A), which may be due to the optics of spinning disk not being compatible with the signal processing algorithms of FasterSTORM (Table S1) [18].

In terms of retention of image intensity information: SOFI performed the best with RainSTORM also producing excellent data, (Figure 3A and C). QuickPALM, GLRT, FasterSTORM, Deconvolution STORM images had punctate and broken appearances in the TIRF and SDSI data. This should not be the case as actin is filamentous as transmission electron microscopy studies have shown [28]. There was a considerable variation in speed of processing, which appeared not to correlate to refinement in resolution accuracy or retention of intensity information (Figure 3C). We found that 3B [16] was unable to reconstruct this size of dataset using a standard lab computer as the algorithm crashed and so excluded it from the analysis. We anticipate that with a multicore image processing cluster that the 3B algorithm would perform admirably. In summary SOFI gave the best compromise between refinement of spatial resolution of the image, retention of image intensity information and convincing image rendering for SDSI data. Therefore for the remainder of studies the SOFI algorithm, as implemented in Localizer was used for SR image processing.

### Comparative study between SDSI and SIM

To examine the validity of the SDSI methodology a parallel study was performed using SIM. SIM differs from stochastic SR methods as the sample is visualised using standard widefield illumination and a structured light pattern is projected onto the sample. Superimposing two or more of these patterns on one another causes an interference pattern (termed moiré pattern), containing harmonic frequencies not available in standard microscopy. Data processing is then carried out to generate an image with resolution of around 120 nm, (Figure S3A) [4], [29]. For both SDSI and SIM imaging specific cellular components of HeLa cells were labelled with fluorescent probes compatible with PALM and STORM methodologies. SIM was shown to be advantageous for visualising structures in 3D as datasets comprising the whole cell could be collected between 2 and 10 minutes, although resolution was limited to 120 nm. SDSI could only be used to visualise structures in a single plane, using currently available image analysis algorithms, as samples bleached during data acquisitions of longer than 10,000 frames.

**Figure 4:**
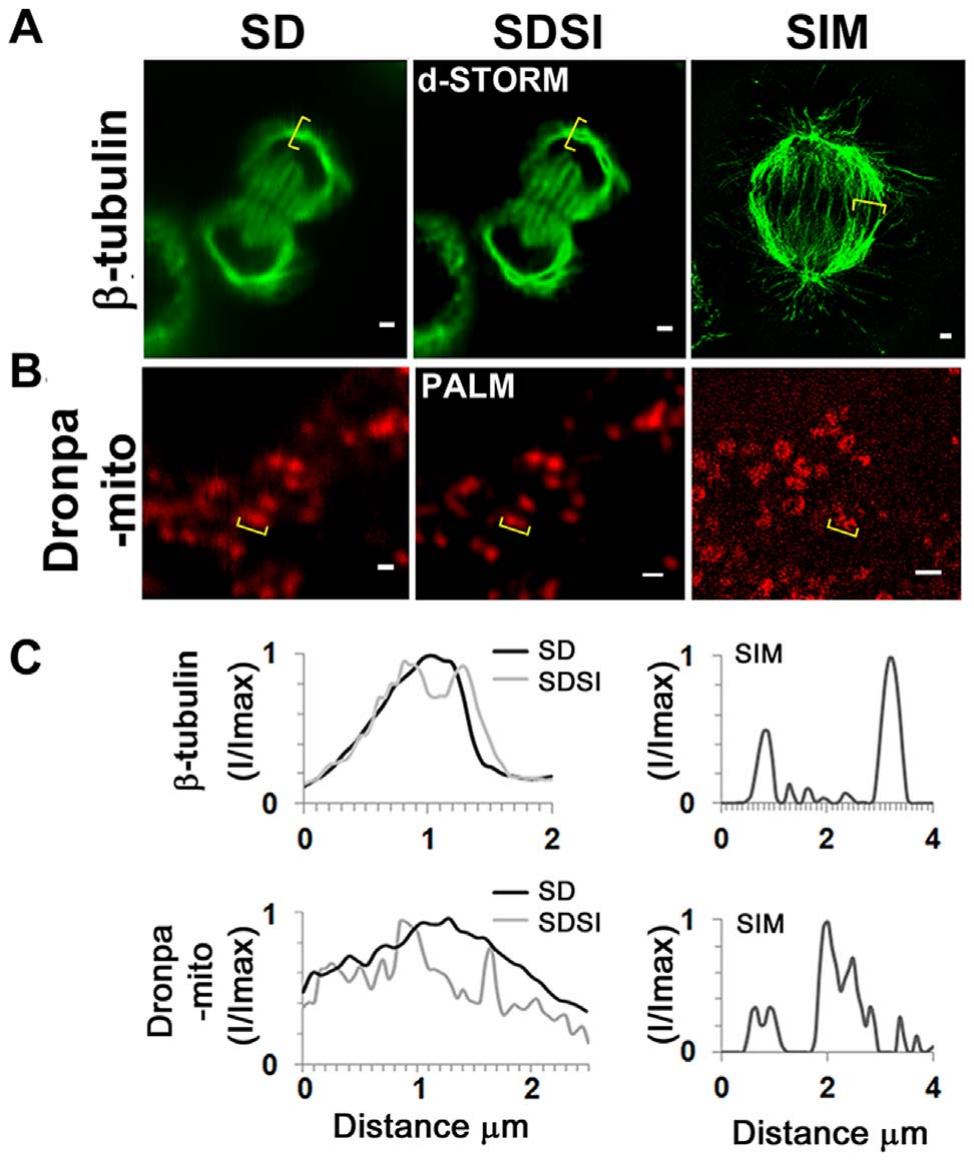
Comparison between single channel SDSI super-resolution imaging and SIM imaging in a medial plane of HeLa cells. (A) Comparison of SD, SDSI-dSTORM and SIM images of the mitotic spindle, the spindle was visualised using β-tubulin antibodies. Secondary Fab fragment antibodies conjugated to AlexaFluor647 were used for dSTORM imaging. SR data was generated using 3^rd^ order SOFI, bar = 1 µm. (B) Comparison of SD, SDSI-dSTORM and SIM images of the mitochondria, the mitochondria were visualised by transfecting cells using the Dronpa-Mito construct for PALM imaging, SR data was generated using 3^rd^ order SOFI, bar = 1 µm. (C) Line-scans (indicated in yellow parenthesis) through the mitotic spindle and mitochondria comparing SD resolution with SDSI and SIM.

Comparing the single plane SDSI image to the SIM showed a resolution enhancement of fine structured microtubules in the mitotic spindle (Figure 4A). Although SIM gave a better 3D reconstructed image of the mitochondria, SDSI images processed using the 3^rd^ order SOFI algorithm showed smaller (80 nm) mitochondria (Figure 4B and C). It was found that Alexa-Fluor dyes gave sufficiently high quantum yields to be used in correlative SIM/STORM microscopy experiments as both had high quantum yield and low photo bleaching. Unfortunately, it was more difficult to carry out correlative PALM/SIM studies due to the fluorescent proteins not being photo-stable enough to withstand the intense illumination required for 3D SIM. Eos-FP probes also performed poorly in SIM studies as they were liable to photo-convert in the process of imaging, generating artefacts of intensity in the output image. However, we found that the Dronpa fluorescent protein performed better than Eos, making it possible to visualise mitochondria (Figure 4B). The 150 nm resolution of Dronpa-Mito in SIM studies was substantially worse than the SIM spindle images generated using Mitotracker-Orange due to the Dronpa-Mito signal being degraded during SIM image acquisition (Figure 4C).

### SDSI of the nucleus

**Figure 5:**
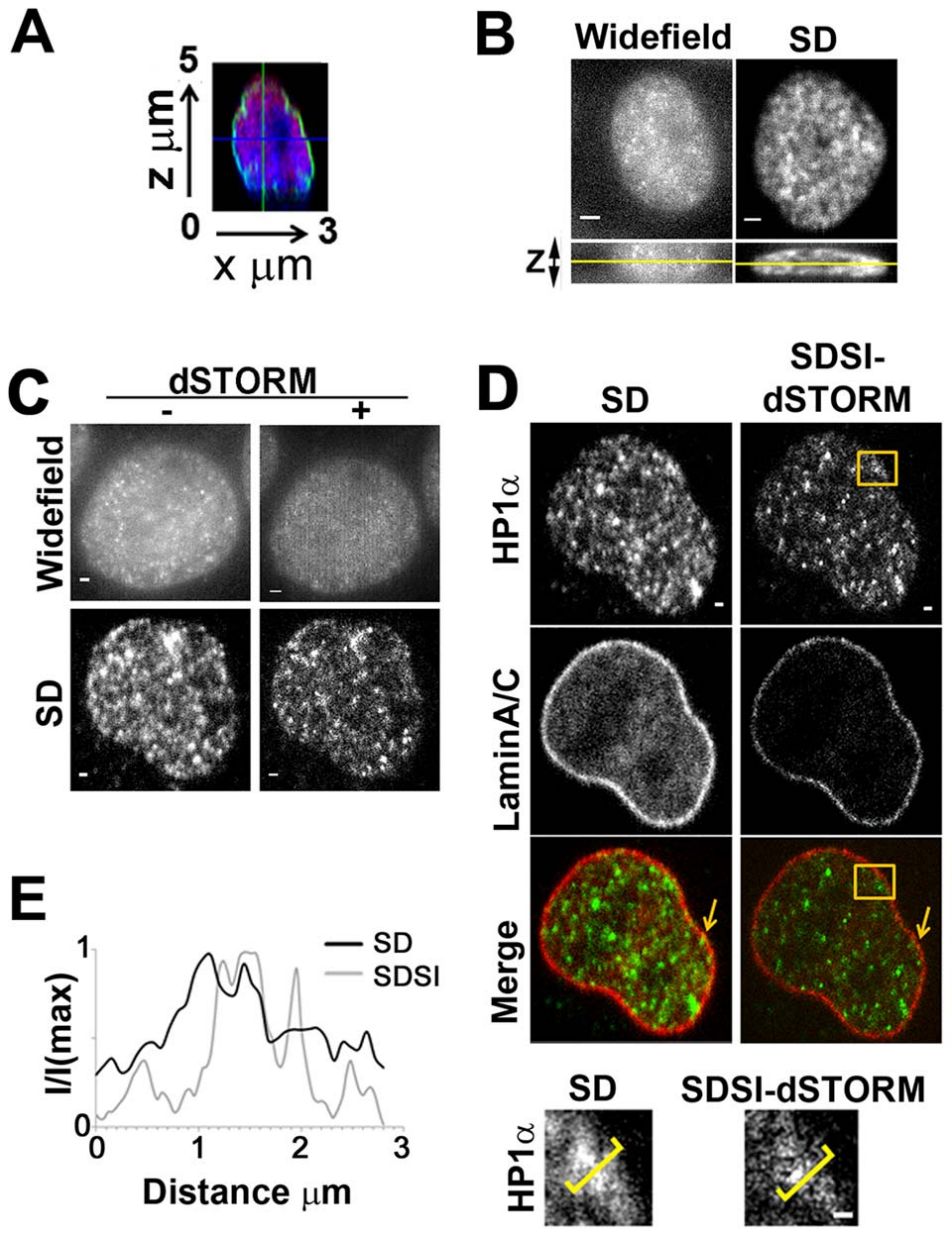
Multispectral super-resolution imaging of the nucleus. (A) Point scanned confocal images showing orthogonal axial (x,z) view of Histone H3 (Red), LaminA/C (Green) and Nuclei (Blue). (B) Wide-field epifluorescent (WF) and spinning disk (SD) x,y and x,z images. Yellow line in x,z images indicates the plane shown in the x,y image of hetero-chromatin in the nucleus visualised using HP1α, bar = 2 µm. (C) dSTORM images of hetero-chromatin in the nucleus visualised using HP1α antibodies acquired by widefield epifluorescence (WF) and spinning disk confocal (SD). SR data from both WF and SD images was generated using 3^rd^ order SOFI, bar = 1 µm. (D) Comparison of SD and SDSI images of hetero-chromatin visualised using HP1α antibodies and the nuclear membrane visualised using LaminA/C antibodies. Secondary FAb fragment antibodies conjugated to AlexaFluor555 and 647 were used for dSTORM imaging, SR data was generated using 3^rd^ order SOFI, bar = 1 µm. Lower panel shows high-resolution region (indicated by orange box in upper panel) of heterochromatin, bar = 1 µm. (E) Intensity profiles, through regions indicated by yellow parenthesis in E, comparing SD resolution with SDSI.

The nucleus is a challenging structure to visualise using current super-resolution methodologies as it is above the maximum lateral height of visualisation of both TIRF (100 nm) and HiLo (500 nm) (Figure 5A). To compare SDSI with widefield epi-illumination (WF) super-resolution we visualised HP1α, a marker of heterochromatin in the nucleus using dSTORM sample preparation. HP1α is present throughout the nucleus and gives punctate staining which can be seen in 3D (Figure 5B). This caused serious problems with WF as the photo-interactions of the labelled HP1α were above and below the plane of focus masked photo-switching in the focal plane (Figure 5B). This meant the SR image processing using the SOFI algorithm was unable to enhance the resolution of WF images or correctly assign structures (Figure 5B). The raw image acquired using the spinning disk system had little out of focus light present as the image was confocal (Figure 5B). Therefore photo-interactions from out of focus light were excluded and the whole image could be accurately processed by SOFI (Figure 5B and C). We found WF imaging of actin, on the basal layer of the cell, gave slightly better data as there are no photo-interactions from out of focus light from below the focal plane (Figure S4A and B). However the resolution improvement with actin was still above the Abbe limit (Figure S4C) whereas SDSI can give super-resolved actin data (Figure 2A).

To show the flexibility of the technique two coloured SDSI was used to resolve hetero-chromatin structures in the medial plane of the nucleus (Figure 5A and D). HP1α and LaminA/C were both prepared using dSTORM sample preparation methodologies, with HP1α being labelled with AlexaFluor555 and LaminA/C with AlexaFluor647, we ensured that the super-resolution sample buffer was compatible with both fluorophores for dSTORM [10]. The heterochromatin fluorescence appeared more diffuse by SD microscopy, while SDSI super-resolution image analysis revealed several small discrete objects ranging from small foci, most likely less than 100 nm in diameter, to larger agglomerations 600 nm in diameter (Figure 5D and E). In addition the background subtraction achieved by SOFI clearly allowed visualisation at high resolution of structures where heterochromatin was absent, such as the nucleolus and PML bodies. SOFI reconstruction revealed regions which appeared as smaller ‘beads’ of heterochromatin whereas confocal microscopy showed only large diffuse staining (Figure 5D and E). Detailed analysis of Lamin A/C also showed variations in the width of the nuclear membrane between 100–200 nm, SIM studies confirmed this observation [30]. Multicolour analysis of our data showed that small puncta of heterochromatin are found immediately adjacent to the nuclear membrane (Figure 5D, indicated by arrow). This is a significant improvement on the confocal data which shows that the nuclear membrane and heterochromatin are localised, which is an erroneous interpretation of the data. Many scientific studies of colocalization could benefit from super-resolution imaging as it provides better information about the precise localization of proteins and obtains stronger and clearer data about protein-protein interactions or their absence.

### Conclusions

We show that multi-spectral SDSI can collect super-resolution images with good S/N, resolved in any selected axial plane within a cell. We compared both PALM and STORM and found that either these techniques can be used either separately or together for generation of super-resolution data. We examined seven different algorithms for processing SD data and found SOFI gave the best retention of image intensity information and provide the most accurate data reconstruction, in terms of spatially assigning all of the emission data found in the original images. This decision was aided by the user friendly GUI for SOFI in the Localizer suite of super-resolution algorithms and the fast data processing times [13]. The speed of processing was important as we intended to use SDSI for imaging multiple large image datasets in 2–3 colours. As SOFI only requires 2000 frames of data for accurate assignment of all of the fluorophore used in this study it would be possible to expand SDSI in future work to encompass applications such as high content screening, imaging of multiple epitopes, and live imaging.

SDSI generated data was comparable to or an improvement on, the SIM dataset. In summary, the SDSI technique was flexible enough to analyse a range of cellular structures in a fast and reliable manner. Our comparative study showed that SIM was more appropriate for imaging 3D structures, such as the mitotic spindle as even the fastest super-resolution data collection and image processing, would still require several hours compared to 10 minutes of SIM imaging. However, PALM/STORM SDSI could generate higher resolved data than SIM for single plane imaging dependant on the image processing algorithm used. Both SIM and PALM/STORM techniques required strong fluorophore labelling and powerful lasers to generate sufficient data for image analysis, due to the signal degradation during data collection and processing.

SDSI is a powerful addition to the range of super-resolution methodologies as it is capable of resolving structures that are located in the centre of the cell where there is a considerable amount of light scattering and densely labelled structures. In the comparative study widefield epi-fluorescence based methodologies could not achieve this (Figure 5B, Figure S4) and TIRF is not able to visualise the nucleus. SDSI has the potential of being applied to visualise multiple epitopes, at high resolution, in the centre of cells instead of cryosectioning or transmission electron microscopy. The increased resolution of SDSI allows accurate interpretation of proteins which are closely connected and are distant neighbours in a disparate group. It also provides an improved assignment of the size and composition of protein-protein complexes. We have shown that multicolour images can be collected and processed in less than two hours using fast image analysis algorithms. This technique provides the utility in multi-parametric biological studies and delivers it to within easy reach of the biomedical researchers who are not microscopy specialists. Naturally if a question demanded higher resolution than 80–100 nm further spatial resolution can be obtained by using other algorithms, although these would require collecting a larger dataset with more photo-interactions for accurate image reconstruction making it impossible to carry out 3D or live imaging studies due to photobleaching/phototoxicity. RainSTORM gave better localization accuracy than SOFI and provided better preservation of image intensity information, in addition it has a useful toolkit for registration of multicolour images [11]. However with our implementation of super-resolution on a spinning disk microscopy and SOFI image processing it would be possible to expand the technique to such applications as live cell imaging, high content screening and 3D imaging. Overall we show that this method presents an encouraging step forward for the wider application of super-resolution methodologies for the biological researcher.

## Materials and Methods

### Cellular sample preparation and transfection

HeLa cells were grown as described elsewhere [29]. For transfection, cells were seeded onto glass-bottomed dishes (WPI, UK) at a density of 2×10^4^ cells ml^−1^ and transfected one day after plating using jetPRIME™ (Polyplus transfection, France) according to the manufacturers protocol with either 1 µg Eos-actin, Eos-H2B or Dronpa-Mito DNA. Cells were incubated for 48 hours after transfection then fixed in 4% Paraformaldehyde diluted from a 16% EM grade stock (Agar Scientific, UK). For STORM/SIM imaging, cells were permeabilized using 0.1× Triton x100 and incubated with primary antibodies raised against β-Tubulin (Sigma, UK), Connexin 43 (BD, UK), LaminA/C (Santa Cruz Biotech, USA), raised against mouse and HP1α (New England Biolabs, USA) raised against rabbit. For STORM imaging samples were incubated with anti—mouse Fab fragment AlexaFluor647 secondary antibodies or AlexaFluor 555 anti-rabbit antibodies (Invitrogen, UK). For SIM imaging anti-mouse Fab fragment AlexaFluor 594 secondary antibodies were used. For SIM F-actin was labelled with Alexa-Fluor488 phalloidin (Invitrogen, UK) and nuclei were labelled using DAPI (Sigma, UK).

### Imaging buffer

For SDSI samples were placed in imaging medium consisting of PBS containing 100 nM 2-mercapto-ethanol, to promote photo-switching, 10 nm ascorbic acid to reduce reactive oxygen species which may damage fluorophores [31] and an oxygen scavenging system [18]. Prior to imaging the medium was degassed by bubbling through nitrogen for 10 minutes. 100 nm gold beads were added to the sample as fiduciary marks (BBI Gold, UK). These are left to settle onto the cells and coverslip for 1 hour prior to imaging and used for post-hoc drift correction and multispectral image registration.

### Spinning disk super-resolution optical setup

The SDSI system was built on an inverted optical microscope (Nikon TE2000E), with a Yokagawa Nipkow spinning disk unit (CSU X1 DSD, Yokagawa Electric Corporation). Four solid state lasers were used as the excitation source: a 405 nm (100 mW), 488 nm (50 mW), 561 nm (50 mW) and 640 nm (100 mW) (Coherent Inc. CA. USA), all lasers were collimated, combined and coupled into an optical fibre (Andor laser combiner, Andor Technologies, UK). The fluorescence emission was filtered using a quad dichroic mirror (Semrock, USA). All imaging was carried out using a 100× 1.4N.A Plan Apochromat VC objective (Nikon, UK). Images were collected on a xIon885 EMCCD camera (Andor Technologies, UK).

For SDSI data was acquired using streaming to the camera, images acquisition rates varied between at 4–6 frames per second. Data was collected using IQ2 software (Andor Technologies, UK). Throughout all SDSI experiments laser power was adjusted to ensure a sparse field of stochastic fluctuations were continuously visible (Figure S2). For SDSI PALM probes were simultaneously activated by a 405 nm (0.5–6% power) and imaged and bleached with either a 488 nm or 561 nm laser (15–30% power). For STORM imaging, the dSTORM methodology was used as described elsewhere [10], For dSTORM imaging 8–20% of the 100 mW power of the 647 nm laser was used. Analysis of fluctuation of intensity of individual actin foci throughout the experiment showed these imaging conditions could capture of photo-conversion events (Figure S2A and B).

### Super-resolution image analysis, including algorithm comparison

Data analysis was carried out on a Dell Alienware PC, 12 GB RAM, Core I5 3.0 GHz quad core processor, 500 GB hard disk. Prior to analysis images were reconstructed and re-registered using FiJi (http://fiji.sc/wiki/index.php/Fiji). To optimise the number of frames for SDSI imaging a 10^5^ frame dataset of 100 nm Tetra speck beads (Invitrogen, UK) was acquired using a 561 nm laser. Data was reconstructed using SOFI algorithms using input datasets of 200, 500, 1000, 2000, 4000, 6000, 8000 and 10,000 frames (Figure S3C). The reconstructed area of the bead was then measured and was repeated for five separate beads. The data analysis showed that a minimum of 2000 frames were required for consistent data reconstruction of the 100 nm beads using SOFI (Figure S3C). An estimate of the localization error along a single axis in the x–y imaging plane showed, for our system, the localization error was 18 nm for single molecule imaging (Materials and Methods). This is due mainly to the small pixel size of our camera, as SDSI detects approximately half the number photo-switching events TIRF does, although individual emission events can be detected (Figure S2A and B and 3B). Using the actin test dataset we found 3^rd^ order SOFI was able to obtain a resolution standard of 80 nm (Figure S3A).For the super-resolution image analysis comparison three datasets were used: A simulated dataset, 64×64 pixel and 800 images with some simulated overlapping emission events, taken from Mukamel et al [17] (Movie S1). A dataset of recombinant actin filaments labelled with AlexaFluor 647 obtained by TIRF microscopy: 64×64 pixels, 5000 frames, which was a kind gift from Dr D. Metcalf, NPL, London UK (Movie S2). A dataset of tdEos-Actin from transfected Hela cells obtained by spinning disk microscopy: 90×90 pixels 5000 frames (Movie S3). 3B and QuickPALM image analysis was carried out using the FiJi plugin [12], [16]. RainSTORM [11] image analysis was carried out in Matlab, (www.mathworks.co.uk). FasterSTORM [18] and DeconSTORM [17] analysis were carried out according to published instructions. The Localizer suite of super-resolution image analysis algorithms [13] was used to conduct SOFI [14] and GLRT [32] image analysis. For the comparative studies each separate test dataset was processed by all of the algorithms and the reconstructed images compiled and measured. A 5000 frame ‘noisy background’ set of images was acquired by using a red fluorescent Perspex slide on the SDSI system to determine if the algorithms would incorrectly assign noise as signal. All data analysis of reconstructed images was carried out using FiJi (http://fiji.sc/Fiji).

### Drift correction

To determine the SDSI system accuracy 100 nm Tetraspeck fluorescent microspheres (Invitrogen, UK) were dried onto glass bottomed dishes (WPI, FL, USA). Bead samples were imaged continuously for 45 minutes and particles tracked using the Particle Tracker plugin in FiJi (http://fiji.sc/wiki/index.php/Particle_Tracker) (Figure S1A–C). Lateral image drift was reduced by placing the system in an environmental enclosure which maintained a stable temperature. To maximise stability the environmental chamber surrounding the system was pre-warmed for 4–6 hours to 32°C prior to imaging for all experiments (Figure 1A and Figure S1). A lateral drift of around 40 nm is assumed for all experiments, however the gold beads are tracked for each experiment and if lateral drift above 40 nm is observed the experiment is rejected and not processed. Axial drift of the objective was corrected using a capacitive feedback system (Pi-Foc 721, Physik Instruments, UK).

### Spinning disk system calibration

PSFs were measured from 100 nm Tetraspeck beads, images were processed by SOFI or QuickPALM and were fitted using the MetroloJ plugin in ImageJ (http://imagejdocu.tudor.lu/doku.php?id=plugin:analysis:metroloj:start) (Figure 1C). The localization accuracy of our system was determined using the standard equation for determination of localization error [6], [19]

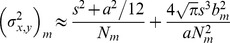
where, s is the standard deviation of the PSF, a is the pixel size in the image (taking into account the system magnification), N_m_ is the total number of photons measured from molecule m, and b_m_ is the number of background photons collected in the fitting window used for molecule m. To determine photon number 5000 frames of data were acquired of td-Eos Actin. Photon number was estimated using FiJi and information from the camera suppliers (http://www.andor.com/learning-academy/count-convert-quantifying-data-in-electrons-and-photons). To determine the resolution achieved by SOFI this dataset was also analysed.

### SIM microscopy

3DSIM was performed on a microscope system (OMX version 2; Applied Precision, USA) as described previously [29]. Raw 3DSIM images were processed and reconstructed using algorithms implemented in SoftWoRx software (Applied Precision, USA) [4], [33], [34].

### Widefield and spinning disk microscopy

Widefield epi-fluorescent microscopy was carried out by removing the spinning disk from the optical path in our system. All other components were identical. For widefield analysis HeLa cells were processed as described above. F-actin was labelled with Alexafluor 555-phalloidin. Spinning disk microscopy images were captured using the same software, laser power, camera gain and exposure time as for SDSI imaging. The widefield and spinning disk images presented are the average of 8 frames of data.

## Supporting Information

Figure S1 **Quantification of axial drift in the super-resolution system.** Drift measurements were obtained from 100 nm Tetraspeck beads (Invitrogen). Imaging conditions were identical to those used in PALM/STORM data acquisition, where data are streamed to the camera at a rate of 4–6 frames per second. Data are pooled from 10 independent tracks, (A) Drift in the x plane in the SDSI system during a 2000 frame data acquisition series. (B) Drift in the y plane in the SDSI system during a 2000 frame data acquisition. (C) Position map showing x, y drift in the SDSI system during a 2000 frame data acquisition.
(TIF)

Figure S2 **The photo conversion properties of Eos-FP Actin.** (A) Images taken from a 5000 frame dataset showing stochastic photo-conversion of Eos-FP actin vesicles. Frame number is indicated on bottom left, data are acquired by streaming at a rate of 4–6 frames per second so total time for dataset acquisition is 1250 seconds approximately, bar = 5 Am. (B) Graph indicating the rate of photo conversion of an individual Eos-Actin vesicle. Imaging conditions were identical to those used in PALM/dSTORM data acquisition, 250 frames of data acquired at a rate of 5 frames per second were measured of a representative sample are shown here.
(TIF)

Figure S3 **Resolution improvement using the SD-SI system.** (A) Graphs comparing the resolution of SSIM and SDSI using an intensity profile of a 100 nm Tetraspeck fluorescent bead. Fluorescence form the bead was excited using a 488 nm laser, Intensity profiles of the bead were collected. Left hand graph shows the OMX SSIM system, right hand side graph compares raw data from the spinning disk with the resolution enhancement in SR images generated using 3rd order SOFI. On both graphs the full width at half maximal intensity are indicated by a line. Data are pooled from 5 separate beads. (B) Graph comparing the number of photo-switching events detected by QuickPALM software in data collected by TIRF and SD. Test datasets of 64×64 images of AlexaFluor647 labeled F-actin (TIRF) and 90×90 pixel images of tdEos-actin in HeLa cells (SD) were compared. (C) Bar chart showing average area reconstructed from SOFI analysis of 100 nm Tetraspeck beads. Datasets of 200, 500, 1000, 2000, 4000, 6000, 8000 and 10000 frames are measured. Data are pooled from 10 separate beads, error bars show standard deviation.
(TIF)

Figure S4 (A) Widefield epifluorescent of HeLa cells labeled with AlexaFlour555 Phalloidin. SR data was generated from a Widefield image set using 3rd order SOFI. (B) High-resolution region (indicated by orange box in image (A) of F-actin bar = 1 Am. (C) Intensity profiles, through regions indicated by yellow parenthesis in E, comparing WF resolution with the super-resolution images generated using 3rd order SOFI.
(TIF)

Table S1 **Table showing how super-resolution algorithms work and any assumptions the algorithms made about the nature of the input image data prior to data processing.**
(DOCX)

Movie S1 **A simulated multiframe fluorescence microscopy data sets in which only a subset of fluorophores was activated in each frame taken from Mukamel et al** [**17**] **.**
(AVI)

Movie S2 **A 5000 frame dataset of AlexaFluor 647 labelled actin generated using TIRF.**
(AVI)

Movie S3 **A 5000 frame dataset of HeLa cells transfected with Eos-Actin generated using SDSI.**
(AVI)
