# Correction: Super-Resolution Imaging Strategies for Cell Biologists Using a Spinning Disk Microscope

**DOI:** 10.1371/annotation/d96769fb-4e7d-4f47-98cf-443447c1471e

**Published:** 2013-12-30

**Authors:** Neveen A. Hosny, Mingying Song, John T. Connelly, Simon Ameer-Beg, Martin M. Knight, Ann P. Wheeler

An affiliation for the second author was incorrectly omitted. In addition to institution number 1, Mingying Song is also affiliated to the following institution: Department of Physics, QMUL, Mile End Road, London E1 4NS, UK. 

