# Supplementary figures and images for: Super-Resolution Imaging Strategies for Cell Biologists Using a Spinning Disk Microscope
Source: PLoS One. 2013 Oct 9;8(10):e74604. doi: 10.1371/journal.pone.0074604 (PMC3793996; doi:10.1371/journal.pone.0074604)

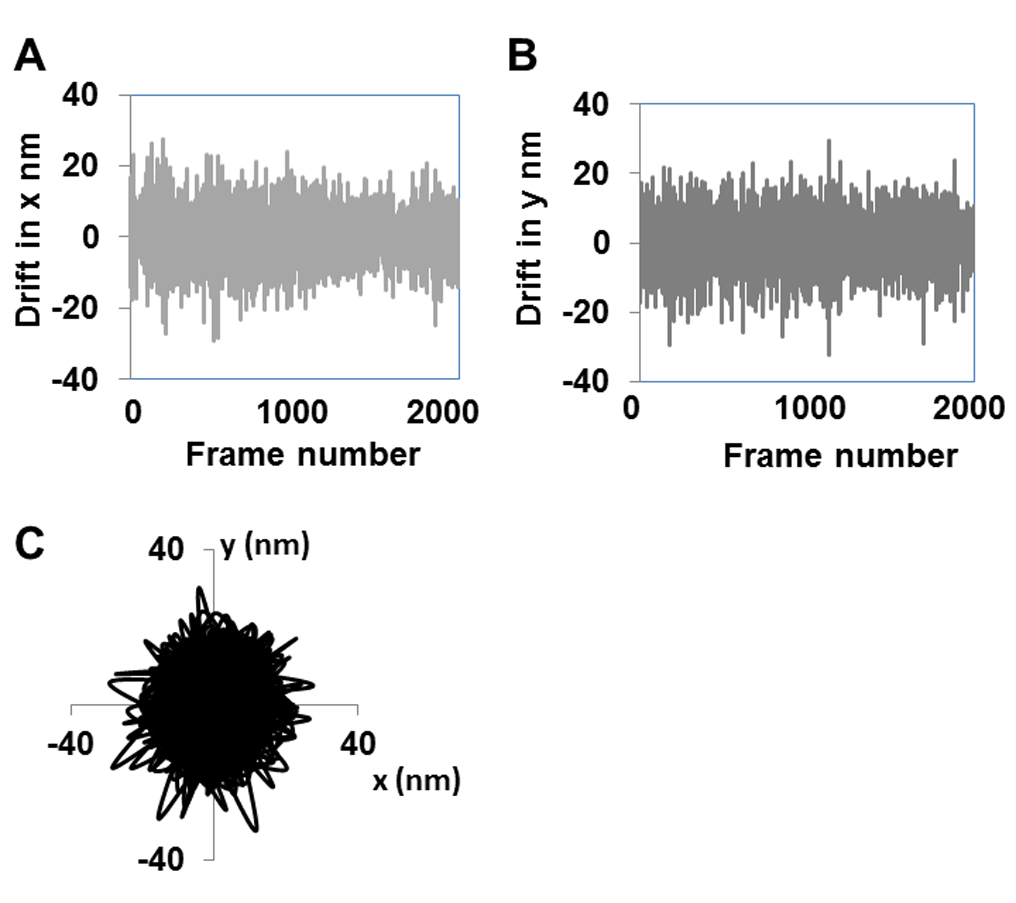

Supplement: Figure S1 — Quantification of axial drift in the super-resolution system. Drift measurements were obtained from 100 nm Tetraspeck beads (Invitrogen). Imaging conditions were identical to those used in PALM/STORM data acquisition, where data are streamed to the camera at a rate of 4–6 frames per second. Data are pooled from 10 independent tracks, (A) Drift in the x plane in the SDSI system during a 2000 frame data acquisition series. (B) Drift in the y plane in the SDSI system during a 2000 frame data acquisition. (C) Position map showing x, y drift in the SDSI system during a 2000 frame data acquisition. (TIF) [file pone.0074604.s001.tif]

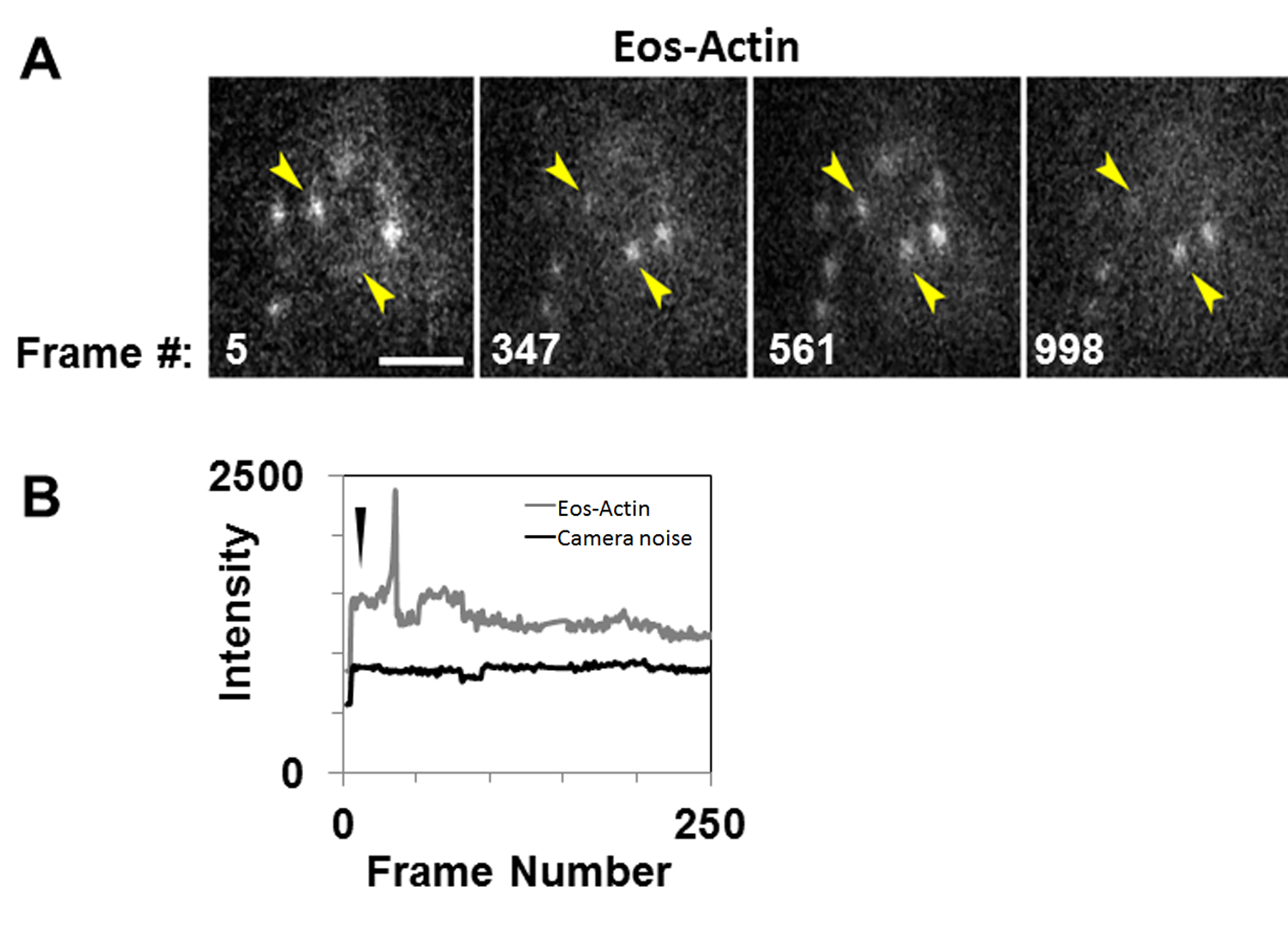

Supplement: Figure S2 — The photo conversion properties of Eos-FP Actin. (A) Images taken from a 5000 frame dataset showing stochastic photo-conversion of Eos-FP actin vesicles. Frame number is indicated on bottom left, data are acquired by streaming at a rate of 4–6 frames per second so total time for dataset acquisition is 1250 seconds approximately, bar = 5 Am. (B) Graph indicating the rate of photo conversion of an individual Eos-Actin vesicle. Imaging conditions were identical to those used in PALM/dSTORM data acquisition, 250 frames of data acquired at a rate of 5 frames per second were measured of a representative sample are shown here. (TIF) [file pone.0074604.s002.tif]

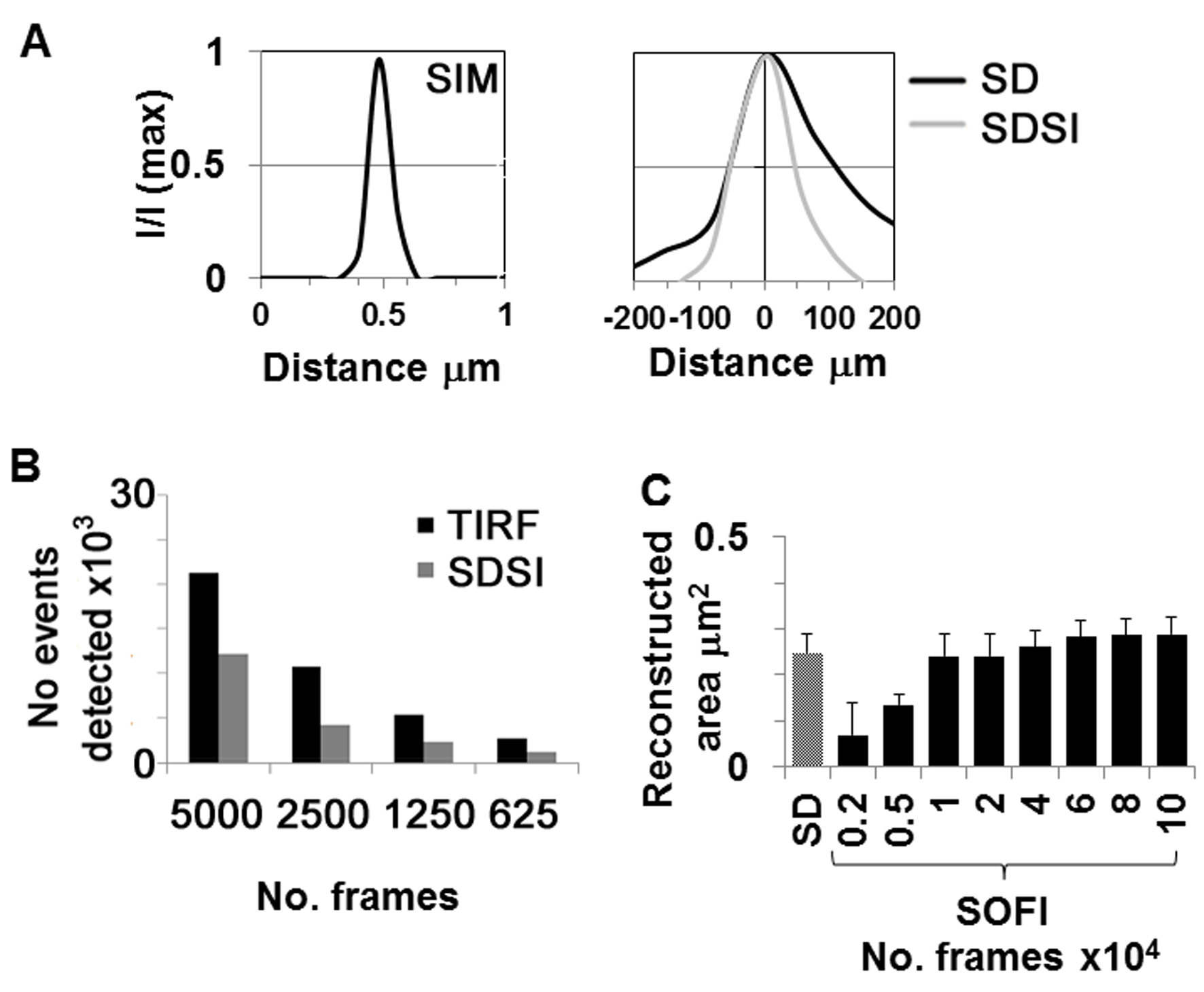

Supplement: Figure S3 — Resolution improvement using the SD-SI system. (A) Graphs comparing the resolution of SSIM and SDSI using an intensity profile of a 100 nm Tetraspeck fluorescent bead. Fluorescence form the bead was excited using a 488 nm laser, Intensity profiles of the bead were collected. Left hand graph shows the OMX SSIM system, right hand side graph compares raw data from the spinning disk with the resolution enhancement in SR images generated using 3rd order SOFI. On both graphs the full width at half maximal intensity are indicated by a line. Data are pooled from 5 separate beads. (B) Graph comparing the number of photo-switching events detected by QuickPALM software in data collected by TIRF and SD. Test datasets of 64×64 images of AlexaFluor647 labeled F-actin (TIRF) and 90×90 pixel images of tdEos-actin in HeLa cells (SD) were compared. (C) Bar chart showing average area reconstructed from SOFI analysis of 100 nm Tetraspeck beads. Datasets of 200, 500, 1000, 2000, 4000, 6000, 8000 and 10000 frames are measured. Data are pooled from 10 separate beads, error bars show standard deviation. (TIF) [file pone.0074604.s003.tif]

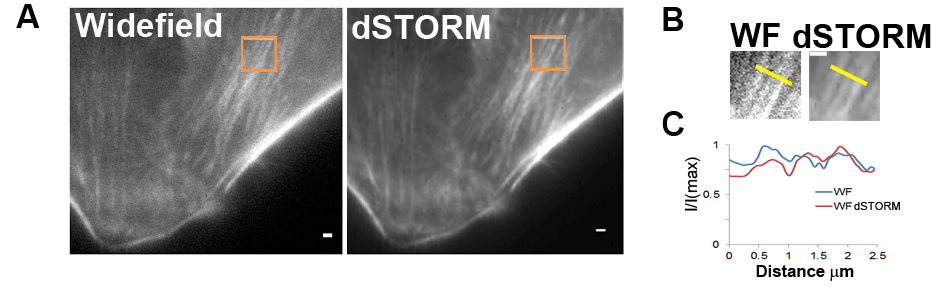

Supplement: Figure S4 — (A) Widefield epifluorescent of HeLa cells labeled with AlexaFlour555 Phalloidin. SR data was generated from a Widefield image set using 3rd order SOFI. (B) High-resolution region (indicated by orange box in image (A) of F-actin bar = 1 Am. (C) Intensity profiles, through regions indicated by yellow parenthesis in E, comparing WF resolution with the super-resolution images generated using 3rd order SOFI. (TIF) [file pone.0074604.s004.tif]
