# Supplementary material for: Super-Resolution Imaging Strategies for Cell Biologists Using a Spinning Disk Microscope
Source: PLoS One. 2013 Oct 9;8(10):e74604. doi: 10.1371/journal.pone.0074604 (PMC3793996; doi:10.1371/journal.pone.0074604)
Supplement: Table S1 — Table showing how super-resolution algorithms work and any assumptions the algorithms made about the nature of the input image data prior to data processing. (DOCX) [file pone.0074604.s005.docx]

| **Algorithm** | **Allows overlapping emitters?** | **How it works** |
| --- | --- | --- |
| **Decon**  **STORM** | Yes | An adjustment of Richardson Lucy deconvolution, which also considers temporal correlations resulting from stochastic emission from individual molecules in PALM/STORM images, can improve resolution fivefold. |
| **Faster STORM** | Partly –small groups of emitters | For each frame; Intensities are assigned to a discrete grid, where grid spacing is smaller than the camera pixel size. This is then convolved against the distribution of intensities in the image and the PSF and intensities spatially assigned. The overall image resolution is determined by both the density of identified molecules. |
| **Quick**  **PALM** | No | Assumes each image is made up of point sources and identifies them. Iteratively assigns localization of molecules using the centroid of intensity. Reconstructs a final image based on position of all the centroids. |
| **Rain**  **STORM** | Partly | Segmentation of each frame per pixel based on intensities, Gaussian fitting of each point, image reconstruction |
| **SOFI** | Yes | Higher-order statistical analysis based on temporal fluctuations in image intensity. A Gaussian correlation function is calculated from intensity fluctuations for each pixel. The SOFI intensity value assigned by integrating intensity with correlation function. |
| **GLRT/**  **Localizer** | No | Assumes image is comprised of individual emitters and assigns centroid on intensity in each frame. Each emitter is put through a generalised likelihood ratio test (deflation) and if it passes it is then Gaussian fitted. |
| **3B** | Yes | Uses Bayesian statistics to fit the position of molecules with fluctuating intensities. The entire time series is globally analysed and the probability map generated takes into account the number of emitters, location and temporal dynamics. |

**Table S1**
